# Safety and metabolic advantages of steroid withdrawal after 6 months posttransplant in de novo kidney transplantation: A 1‐year prospective cohort study

**DOI:** 10.1002/iid3.576

**Published:** 2021-12-15

**Authors:** Jun B. Bang, Chang‐Kwon Oh, Yu S. Kim, Sung H. Kim, Hee C. Yu, Chan‐Duck Kim, Man Ki Ju, Byung J. So, Sang Ho Lee, Sang Y. Han, Cheol W. Jung, Joong K. Kim, Hyung J. Ahn, Su H. Lee, Ja Y. Jeon

**Affiliations:** ^1^ Department of Surgery Ajou University School of Medicine Suwon South Korea; ^2^ Department of Transplantation Surgery, Research Institute for Transplantation Yonsei University College of Medicine Seoul South Korea; ^3^ Department of Surgery, Wonju Severance Christian Hospital Yonsei University Wonju College of Medicine Wonju South Korea; ^4^ Department of Surgery Jeonbuk National University College of Medicine Jeonju South Korea; ^5^ Department of Internal Medicine, Kyungpook National University Kyungpook National University Hospital Daegu South Korea; ^6^ Department of Surgery Yonsei University College of Medicine Seoul South Korea; ^7^ Department of Surgery Wonkwang University Hospital Iksan South Korea; ^8^ Department of Internal Medicine Kyung Hee University Seoul South Korea; ^9^ Department of Internal Medicine Inje University Ilsan Paik Hospital Goyang South Korea; ^10^ Department of Surgery Korea University College of Medicine Seoul South Korea; ^11^ Department of Internal Medicine Bong Seng Memorial Hospital Busan South Korea; ^12^ Department of Surgery Kyung Hee University School of Medicine Seoul South Korea; ^13^ Department of Endocrinology and Metabolism Ajou University School of Medicine Suwon South Korea

**Keywords:** cholesterol, glucose tolerance test, kidney transplantation, steroids

## Abstract

**Introduction:**

This prospective multicenter study aimed at investigating the safety and metabolic advantages of steroid withdrawal (SW) therapy in kidney transplant recipients with tacrolimus–mycophenolate mofetil‐based immunosuppression.

**Methods:**

We analyzed 179 recipients who received kidney transplantation from March 2016 and September 2018. In 179 recipients, 114 patients maintained an immunosuppressive regimen including steroids (steroid continuation [SC] group). The remaining 65 patients were determined to withdraw steroid therapy after 6 months posttransplant (SW group). Metabolic parameters and graft functions of the two groups were evaluated.

**Results:**

The estimated glomerular filtration rates at 12 months posttransplant were 67.29 ± 20.29 ml/min/1.73 m^2^ in SC group and 73.72 ± 17.57 ml/min/1.73 m^2^ in SW group (*p* < .001). The acute rejection occurred to four recipients in the SC group (3.5%) and no acute rejection occurred to SW group recipients during the 6–2 months posttransplant period. Oral glucose tolerance tests revealed that recipients in the SW group were more improved in glucose metabolism than the SC group during 6–12 months posttransplant. In addition, cholesterol levels and blood pressure decreased after the withdrawal of steroids in the SW group.

**Conclusion:**

In conclusion, a 6‐month withdrawal of steroids in recipients with low immunological risk and stable graft function can be safely conducted and result in improvement of metabolic profiles. Stable recipients without biopsy‐proven acute rejection and proteinuria can safely withdraw from steroids out of a maintenance immunosuppressive regimen 6‐months posttransplant. A long‐term follow‐up study is needed to verify our results.

## 1 INTRODUCTION

Kidney transplantation (KT) is the most effective treatment modality for patients with end‐stage renal disease.^1^ Many immunosuppressive regimens have been introduced to improve graft survival. Among the many regimens, that consisting of tacrolimus, mycophenolate mofetil (MMF), and steroids have been the most commonly used as maintenance treatment in patients who have undergone KT for better graft survival and fewer acute rejection episodes.^2^

Steroids, which are commonly used as maintenance therapy, are also effective at inhibiting acute rejection. However, long‐term use of steroids carries many side effects, including hypertension, hyperlipidemia, impaired glucose metabolism, new‐onset diabetes after transplantation (NODAT), weight gain, infection, and osteoporosis.^3^, ^4^, ^5^ These side effects affect patient quality of life and adversely affect patient survival. With limited evidence on calcineurin inhibitor withdrawal, reducing and withdrawing steroids have become attractive ways to reduce immunosuppression and steroid‐induced side effects.^6^ Several studies have proven the safety and efficacy of steroid withdrawal (SW) with a regimen consisting of tacrolimus and MMF.^7^, ^8^ However, the timing of SW and the criteria of target patients who would benefit from SW are controversial.^7^, ^9^, ^10^, ^11^

In a previous randomized controlled study, early SW therapy was associated with an increase in biopsy‐proven acute rejection (BPAR) compared to steroid maintenance therapy in patients undergoing tacrolimus and an MMF‐based regimen.^12^ According to Haller et al.,^13^ SW earlier than 18 months posttransplant is associated with an increased risk of allograft loss. In addition, grafts in patients at high immunological risk are more vulnerable to an SW regimen.^14^ In a very recent study, early SW in deceased‐donor KT recipients with delayed graft function led to a worse graft failure rate.^9^

Overall, many findings have led us to conclude that SW therapy should be introduced safely to a select patient group at low immunological risk, with stable graft function, and no previous BPAR.^15^ Nevertheless, these controversies have made it difficult to manage SW; there are many advantages of an SW regimen in terms of reducing the cardiovascular risk that outweighs the increased risk of steroid‐sensitive BPAR.^16^ In this multicenter prospective cohort study, we investigated the safety and metabolic advantages of SW therapy in KT recipients taking a tacrolimus‐MMF‐based immunosuppressive regimen. Notably, we evaluated the extent to which SW affected glucose and insulin metabolism using the oral glucose tolerance test (OGTT).

## 2 METHODS

### 2.1 Study population

This multicenter prospective cohort study was performed between March 2016 and September 2018. Adult KT recipients with ages ranging from 20 to 65 were included in this study. The recipients with the following conditions were excluded: multiple organ transplants or double KT or organs donated after cardiac death; previously organ transplanted recipients; ABO‐incompatible recipients; positive complement‐dependent cytotoxic cross‐matching recipients; and history of malignancy in the previous 5 years. After exclusion, a total of 222 recipients consisted of the eligible population. Among these recipients, 27 recipients were excluded due to immunosuppression protocol violation, 12 recipients were excluded because of MMF withdrawal resulting from drug side effects, two recipients were follow‐up loss, and two recipients had a graft loss within the first month after transplantation. After all, remained 179 recipients finally were included in the study population. The informed consent was provided to all patients, and the independent Institutional Review Board of each center approved this study protocol (AJIRB‐MED‐CT4‐15‐422). This study was performed in accordance with the World Medical Association Declaration of Helsinki Ethical Principles.

### 2.2 Immunosuppression and grouping by SW protocol

The same immunosuppressive protocol was applied to each transplant center. The immunosuppressive regimen consisted of basiliximab (Simulect®; Novartis) as induction therapy, tacrolimus (TacroBell®; Chong K. Dang), MMF (MY‐REPT®; Chong K. Dang), and corticosteroids. Basiliximab was administered just before transplantation and 4 days after transplantation. Tacrolimus was initiated 2 days before KT with an initial dose of 0.05–0.1 mg/kg. The target trough level of tacrolimus was between 5 and 12 ng/ml until posttransplant 3 months and then the target level was downward adjusted between 3 and 8 ng/ml until the follow‐up period finished.

**Figure 1:**
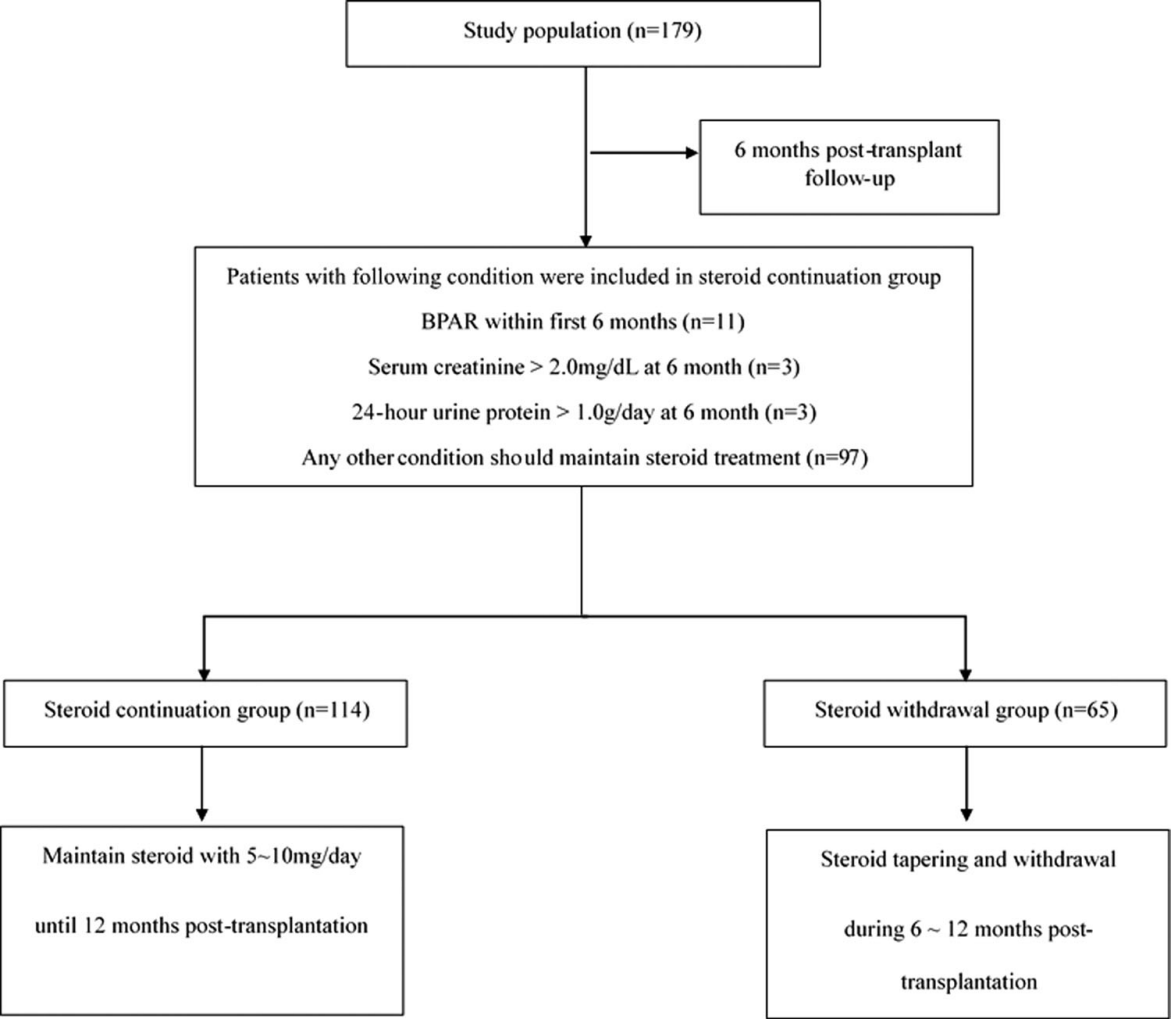
Patient's flowchart. BPAR, biopsy‐proven acute rejection

Steroids were administrated intravenously at 500 mg on the day of transplantation, 250 mg on the day after transplantation, and were gradually tapered to a maintenance dose of more than 5 mg a day until posttransplant 6 months. At 6 months posttransplant, the recipients with the following conditions should maintain steroid therapy until 12 months posttransplant: any BPAR within the first 6 months; serum creatinine >2.0 mg/dl at 6 months; 24‐h urine protein >1.0 g/day at 6 months. In the recipients who did not show any conditions described above, the clinicians judged to decide whether recipients should maintain steroid treatment or not based on the clinician's judgment. After assessments, 114 recipients remained as steroid continuation group (SC group) and 65 recipients were classified as SW group (SW group) (Figure 1). In the SC group, steroid was administrated until 1‐year after transplantation. In the SW group, the steroid was withdrawn after 6 months posttransplant and the withdrawal was maintained until the follow‐up period ended. MMF was started within 72 h after transplantation at a dosage of 1.0–2.0 g per day. For patients experiencing leukopenia or gastrointestinal toxicity, MMF dose was reduced according to a defined protocol and was guided by the clinical severity and course of the adverse event. More than 14 consecutive days of MMF withdrawal were referred to as a protocol violation.

### 2.3 Assessments of graft function and metabolic parameters

Regular patient visits were scheduled on the day before transplantation (baseline) and every 3 months until 1‐year posttransplant. A full physical examination and routine laboratory results with the tacrolimus level were evaluated at every regular visit. Renal function was assessed using the serum creatinine level, the estimated glomerular filtration rate (eGFR), and the chronic kidney disease‐epidemiology collaboration equation.^17^ In this study, no protocol renal biopsy protocol was performed. When acute rejection was clinically suspected, ultrasound‐guided renal biopsies were performed using a 16‐G needle. The diagnosis of acute rejection was classified according to the Banff classification criteria.^18^

Blood pressure was checked and laboratory results including lipid profiles were collected from all participants at every visit. The need to treat hypertension and dyslipidemia was judged by the treating clinician. In addition, the OGTT was performed pretransplantation and every 3 months after the transplant to evaluate the incidence of NODAT, changes in fasting glucose level, 2 h glucose level, and insulin sensitivity. All OGTT procedures were performed according to the American Diabetes Association guidelines.^19^ NODAT was diagnosed if one of the following conditions existed: fasting plasma glucose ≥126 mg/dl; 2 h plasma glucose level ≥200 mg/dl during the OGTT; hemoglobin A1c (HbA1c) level >6.5%; and requirement of an oral hypoglycemic agent or an insulin injection. Patients with prediabetes were defined by the presence of impaired fasting glucose and/or impaired glucose tolerance, a fasting plasma glucose of 100–125 mg/dl, and 2 h plasma glucose during the OGTT of 140–199 mg/dl, respectively. Once diagnosed with NODAT, the patient was treated with insulin or an oral hypoglycemic agent. The OGTT‐derived insulin sensitivity index for transplantation (ISITX) was used to estimate insulin sensitivity.^20^, ^21^ Comparative analyses of all metabolic parameters were performed through the posttransplant period to evaluate the effectiveness of SW.

### 2.4 Statistical analysis

For categorical variables, data were expressed as a number of patients and a percentage of derived groups, analyzed by Pearson's *χ* ^2^ test and Fisher's exact test. Continuous variables were expressed as a mean ± standard deviation and analyzed by using the Student's *t*‐test and Mann–Whitney test. An analysis of the effects of SW, paired *t*‐test, or Wilcoxon rank‐sum test was used. The *p *< .05 was considered significant. Data analysis was operated by using SPSS version 20.0 (SPSS Inc).

## 3 RESULTS

### 3.1 Basic characteristics of the study population

The mean age of the 179 study participants was 48.2 ± 10.1 years, and 116 (64.8%) were male. Hypertensive nephropathy was the most common cause of end‐stage renal disease in the total population (*n* = 42, 23.5%). The number of recipients with pretransplant diabetes mellitus (DM) was 81 (45.3%), which included 33 recipients with unrevealed DM discovered by a pretransplant OGTT. The mean age of the donors was 43.7 ± 13.6 years, and 95 (53.1%) were male. A total of 113 (63.1%) living donor transplantations were performed, and 66 deceased donor transplantations were performed (36.9%).

The study population was divided into the SC and SW groups. The details of the characteristics are described in Table 1. The mean age of the recipients was not significantly different between the two groups (48.4 ± 11.1 vs. 47.8 ± 8.0 years). Eighty (70.2%) male recipients were in the SC group and 36 (55.4%) were in the SW group (*p* = .052). The dialysis duration of the SW group was significantly longer than that in the SC group (*p* = .002). The mean donor ages were 44.6 ± 13.1 years in the SC group and 42.1 ± 14.3 years in the SW group. Living donor transplantation was more frequent than a deceased liver donation in the SC group (74.6% vs. 43.1%, *p* < .001).

**Table 1:** Basic characteristics of the study population

	Steroid continuation (*n* = 114)	SW (*n* = 65)
Recipients variables		
Age (y)	48.4 ± 11.1	47.8 ± 8.0
Male gender	80 (70.2%)	36 (55.4%)
BMI (kg/m^2^)	23.5 ± 3.7	24.0 ± 3.7
Cause of ESRD*		
Hypertension	36 (31.6%)	6 (9.2%)
Glomerulonephritis	30 (26.3%)	11 (16.9%)
DM	23 (20.2%)	17 (26.2%)
Polycystic kidney disease	5 (4.4%)	3 (4.6%)
Other	3 (2.6%)	0
Unknown	17 (14.9%)	28 (43.1%)
DM at pretransplantation^a^	55 (48.2%)	26 (40%)
Dialysis modality		
Hemodialysis	56 (49.1%)	38 (58.5%)
Peritoneal dialysis	9 (7.9%)	16 (24.6%)
Preemptive transplantation	49 (43%)	11 (16.9%)
Dialysis duration (month)*,^b^	3 (0–31.5)	40 (4–80)
PRA positivity at transplantation		
Class I	9 (7.9%)	6 (9.2%)
Class II	7 (6.1%)	6 (9.2%)
HLA mismatches		
0–1	23 (20.2%)	8 (12.3%)
2–4	64 (56.1%)	43 (66.1%)
5–6	27 (23.7%)	14 (21.6%)
Donor variables		
Age (y)	44.6 ± 13.1	42.1 ± 14.3
Male	56 (49.1%)	39 (60%)
Type of donation*		
Living	85 (74.6%)	28 (43.1%)
Deceased	29 (25.4%)	37 (56.9%)

*Note*: The continuous variable was expressed by mean ± standard deviation and the number of cases with percentages was for the categorical variables. Abbreviations: BMI, body mass index; DM, diabetes mellitus; ESRD, end‐stage renal disease; HLA, human leukocyte antigen; PRA, panel reactive antibody.

^a^ DM was diagnosed by a history of DM medication and pretransplant oral glucose tolerance test results.

^b^ Median (interquartile range).

* *p* < .05.

### 3.2 Posttransplant clinical parameters in the groups

Table 2 shows the clinical parameters of the SC and SW groups at 6 and 12 months. Tacrolimus dose, tacrolimus trough level, and steroid dose of the SW group were significantly lower than those of the SC group at 6 months (*p* = .010, *p* = .027, and *p *< .001, respectively). The mean MMF dose at 6 months was similar between the two groups. The mean serum creatinine level was significantly lower in the SW group than that in the SC group (1.25 ± 0.44 vs. 1.00 ± 0.25, *p* < .001). In addition, the eGFR values at 6 months were 67.75 ± 20.55 ml/min/1.73 m^2^ in the SC group and 80.08 ± 16.13 ml/min/1.73 m^2^ in the SW group (*p* < .001). As a result, the SW group had better graft function than the SC group. No graft loss or patient death was experienced in either group during the first 6 months. BPAR occurred in 11 recipients (9.6%) during the first 6 months, and these recipients were included in the SC group following the criteria.

**Table 2:** Comparison of posttransplantation clinical parameters between two groups

	At 6 months			At 12 months		
	SC group (*n* = 114)	SW group (*n* = 65)	*p*	SC group (*n* = 114)	SW group (*n* = 65)	*p*
Tacrolimus dose (mg/day)	3.5 ± 2.0	2.6 ± 1.1	.001	3.2 ± 2.0	2.3 ± 1.0	<.001
Tacrolimus trough level (ng/ml)	5.8 ± 2.0	5.1 ± 2.3	.027	6.3 ± 3.1	5.4 ± 2.7	.066
MMF dose (mg/day)	1159.1 ± 258.2	1171.9 ± 274.1	.755	1142.5 ± 268.0	1114.6 ± 299.8	.523
Steroid dose (mg/day)	8.3 ± 5.5	4.7 ± 1.4	<.001	7.2 ± 3.4	0	<.001
Serum creatinine (mg/dl)	1.25 ± 0.44	1.00 ± 0.25	<.001	1.26 ± 0.42	1.10 ± 0.33	.010
eGFR (ml/min/1.73 m^2^)^a^	67.75 ± 20.55	80.08 ± 16.13	<.001	67.29 ± 20.29	73.72 ± 17.57	.034
BPAR	11 (9.6%)	0	.008	4 (3.5%)	0	.298
Graft loss or patient death	0	0		0	0	

Abbreviations: BPAR, biopsy‐proven acute rejection; eGFR, estimated glomerular filtration rate; MMF, mycophenolate mofetil; SC, steroid continuation; SW, steroid withdrawal.

^a^ Chronic kidney disease‐epidemiology collaboration method.

The mean dose of tacrolimus was significantly different between the two groups at 12 months. The mean dose of steroid was zero in the SW group and 7.2 ± 3.4 mg in the SC group (*p* < .001). The serum creatinine level was lower in the SW group than that in the SC group (*p* = .010); therefore, the eGFR was higher in the SW group (*p* = .034). No graft loss or patient death occurred until 12 months posttransplant. BPAR occurred in four recipients in the SC group during Months 6–12.

### 3.3 Incidence of NODAT and variables derived from the OGTT

**Figure 2:**
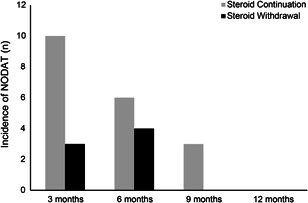
The incidence of new‐onset diabetes after transplantation (NODAT) during the follow‐up period in two groups

The incidence of NODAT was evaluated in 3‐month intervals using OGTT (Figure 2). The cumulative incidence of NODAT during Year 1 was 26.5% (*n* = 26) and the absolute incidence of NODAT at 1 year was 6.1% (*n* = 6). Sixteen recipients were diagnosed with NODAT in the SC group (14.0%), and seven recipients were diagnosed with NODAT in the SW group (10.8%) before 6 months posttransplant. Only three recipients in the SC group were diagnosed with NODAT 9 months posttransplant (2.6%). No cases of NODAT occurred in the SW group during Months 6–12.

**Figure 3:**
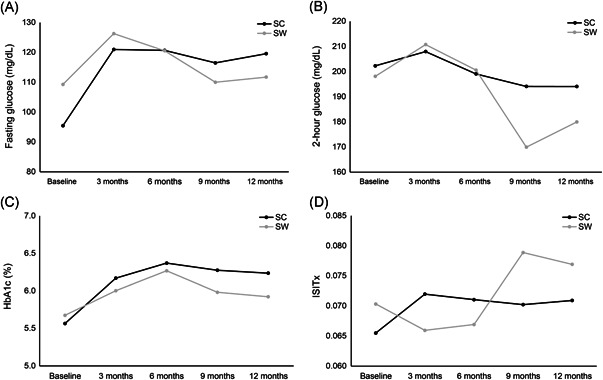
The comparison of glucose metabolism parameters between steroid continuation (SC) and steroid withdrawal (SW) groups

Figure 3 shows the 1‐year follow‐up of fasting glucose level, 2 h glucose level, HbA1c, and ISITX. Until 6 months posttransplant, there was no significant difference in these variables between the two groups. Fasting glucose level was significantly lower in the SC group than that in the SW group 9 months posttransplant (116.5 ± 31.0 vs. 109.9 ± 30.0, *p* = .049). No noticeable change in fasting glucose level occurred in the SC group at 12 months compared to 6 months (*p* = .752). However, a significant change in fasting glucose level was detected in the SW group at 12 months compared to 6 months (*p* = .005). The 2 h glucose level showed similar results as those of fasting glucose level; a significant 6‐month interval change was observed in the SW group, but not in the SC group (*p* = .001 and *p* = .792). The HbA1c level was significantly different between the Groups 9 and 12 months posttransplant (6.27 ± 1.20 vs. 5.98 ± 1.04, *p* = .044 and 6.23 ± 1.10 vs. 5.92 ± 1.13, *p* = .006, respectively). A significant change in HbA1c was observed in the SW group from 6 to 12 months posttransplant (*p* < .001). The ISITX value of the SW group was significantly higher than that of the SC group at 9 months posttransplant (*p* = .004). The interval change from 6 to 12 months was significant in the SW group but not in the SC group (*p* = .001 and *p* = .565, respectively).

### 3.4 Change in metabolic parameters after SW

The metabolic parameters and the incidence of treated hypertension and hypercholesterolemia were assessed after SW had begun 6 months posttransplant. The metabolic parameters 12 months posttransplant generally improved in the SC and SW groups compared to those at 6 months posttransplant (Table 3). Total cholesterol, high‐density lipoprotein (HDL), low‐density lipoprotein (LDL), and systolic and diastolic blood pressures significantly decreased in the SW group at 12 months compared to 6 months. By contrast, only total cholesterol significantly decreased during Months 6–12 in the SC group. Body mass index (BMI) and body weight did not change significantly in either group. The mean differences in total cholesterol, HDL, and LDL in the SW group were significantly larger than those in the SC group between 6 and 12 months. Bodyweight, BMI, and systolic and diastolic pressures were not significantly different between the groups. Nineteen patients (16.7%) in the SC group were treated for hypertension and 35 (30.7%) were treated for hypercholesterolemia. Twelve recipients (18.5%) were treated for hypertension, and 16 recipients (24.6%) were treated for hypercholesterolemia in the SW group. No significant differences in the number of patients treated for hypertension and hypercholesterolemia were observed between the two groups.

**Table 3:** Metabolic parameters between 6 and 12 months posttransplantation in two groups

	SC group			SW group			*p*
Characteristics	6 months	12 months	Δ 6–12 months	6 months	12 months	Δ 6–12 months	
Total cholesterol (mg/dl)	184.5 ± 37.4	172.5 ± 32.2	−11.3 ± 35.2*	196.1 ± 36.1	165.0 ± 32.6	−31.1 ± 34.5**	.000***
HDL(mg/dl)	60.3 ± 18.0	58.2 ± 18.9	−2.4 ± 13.7	67.3 ± 18.0	51.5 ± 14.3	−16.3 ± 13.7**	.000***
LDL (mg/dl)	98.8 ± 29.7	92.8 ± 32.3	−5.4 ± 32.9	106.4 ± 31.2	88.3 ± 29.6	−18.4 ± 28.5**	.010***
Body weight (kg)	63.3 ± 10.6	63.8 ± 10.9	0.7 ± 2.6	63.7 ± 12.2	63.9 ± 12.6	0.3 ± 2.2	.270***
BMI (kg/m^2^)	22.9 ± 3.0	23.1 ± 3.2	−0.03 ± 5.85	23.5 ± 3.5	23.5 ± 3.2	0.41 ± 4.17	.640***
Systolic blood pressure (mmHg)	127.2 ± 15.1	124.3 ± 13.0	−4.0 ± 26.8	129.6 ± 15.3	125.7 ± 13.5	−3.9 ± 12.5	.966***
Diastolic blood pressure (mmHg)	79.7 ± 11.9	76.6 ± 10.7	−3.8 ± 17.4	84.3 ± 14.5	80.5 ± 11.0	−3.8 ± 9.3	.998***
Treated hypertension	19 (16.7%)			12 (18.5%)			.455
Treated hypercholesterolemia	35 (30.7%)			16 (24.6%)			.491

Abbreviations: BMI, body mass index; HDL, high‐density lipoprotein; LDL, low‐density lipoprotein; SC, steroid continuation; SW, steroid withdrawal.

* Wilcoxon rank‐sum test showed *p* < .05 between 6 and 12 months in the SC group.

** Wilcoxon rank‐sum test showed *p* < .05 between 6 and 12 months in the SW group.

*** *p*‐value for the data of Δ 6–12 months between SC and SW group by Mann–Whitney test.

## 4 DISCUSSION

In this multicenter prospective study, we evaluated the safety and metabolic advantages of SW 6 months after KT in low immunological risk recipients. The results suggested that steroid therapy could be safely withdrawn 6 months posttransplant in low immunological risk recipients who had stable graft function during the first 6 months posttransplant. No BPAR was observed after withdrawal of the steroid, which occurred in four recipients during Months 6–12 posttransplant in those who continued steroid use. The metabolic variables improved after SW although the follow‐up period of the withdrawal was only 6 months. No NODAT was observed in SW group recipients, whereas three recipients experienced NODAT in the SC group during Months 6–12 posttransplant.

Many studies have evaluated the effects of avoidance or withdrawal of steroids because long‐term steroid use can induce undesirable side effects. A recent Austrian cohort study reported that SW is associated with an increased risk of graft loss within 18 months posttransplant, although a cyclosporine‐based immunosuppressive regimen, which was not applied in our study, was used in half of the study population.^13^ Desai et al.^22^ reported that SW strategies between 6 and 12 months posttransplant reduce adverse cardiovascular events with no worsening of BPAR or graft loss rate. In another randomized prospective study, recipients taking a tacrolimus‐based 6‐month SW regimen had a BPAR rate of 4.8% between the 6‐ and 12‐month follow‐up visits.^23^ A recent multicenter randomized controlled study showed that SW 7 days posttransplant provides similar long‐term graft survival and function compared to SC, and provides improved cardiovascular risk factors, such as NODAT, weight gain, and triglycerides.^12^ In that study, recipients with SW had a lower peak panel reactive antibody, shorter cold ischemic time, and younger donor age without acute rejection during the first‐week posttransplant. Presumably, SW could be performed early with low immunological risk and good graft function. The Kidney Disease: Improving Global Outcomes guidelines also suggest that steroids can be discontinued during the first week after transplantation in patients who are at low immunological risk and who receive induction therapy.^24^

In our study, we established the SW criteria that recipients should maintain stable graft function and have low immunological risk. The conditions that should maintain steroid therapy in this study included BPAR within the first 6 months, serum creatinine >2.0 mg/dl at 6 months, and 24 h urine protein >1.0 g/day at 6 months. At least 6 months of steroid maintenance was needed to provide sufficient safety for SW because acute rejection frequently occurs in KT recipients within 6 months posttransplant. We assumed that there would be little change in BPAR rate in the future if no BPAR occurred during the first 6 months posttransplant. In our data, 65 recipients in the SW group did not experience BPAR at 1 year with stable graft function. Furthermore, the serum level of creatinine and eGFR value were better at 1 year in the SW group than in the SC group. SW was available only for those who had low serum creatinine and no BPAR until 6 months posttransplant. Therefore, in a way, the recipients with more immunosuppressive agents paradoxically seemed to have lower graft function than the SW group, because the recipients in the SC group already had a BPAR. In addition, clinicians tended to avoid choosing the recipient with worse graft function into the SW group. Consequentially, we thought that the recipients in the SC group were inevitably more vulnerable to developing BPAR than the SW group recipient.

A recent retrospective study by Wehmeier et al.^25^ suggested that surveillance biopsies 3 and 6 months posttransplant are useful to determine the SW strategy by detecting subclinical acute rejection. A graft biopsy provides pathological evidence of acute rejection; however, performing a biopsy could be a disadvantage to recipients with stable graft function because of the bleeding risk and economic burden. Therefore, a sufficient steroid maintenance period would provide a safe withdrawal strategy without a protocoled biopsy.

In this study, we evaluated NODAT and glucose metabolism defined by the 3‐month OGTT. After excluding pre‐existing diabetes with the baseline pretransplantation OGTT, 26 recipients were diagnosed with NODAT during the study period. NODATs were discovered in both groups during the first 6 months posttransplant. Only SC group recipients were diagnosed with NODAT after 6 months. Notably, no significant relationship was detected between the development of NODAT and SW in this study because the incidence of NODAT is high during the early posttransplant stage.^26^ The OGTT results of this study suggest that SW improved glucose tolerance, insulin sensitivity, and HbA1c level. Glucose and 2 h plasma glucose significantly decreased in the SW group during Months 6–12 posttransplant, but not in the SC group. Insulin sensitivity improved after 6 months posttransplant in the SW group. This result can be explained by a previous study reporting that insulin sensitivity is primarily associated with steroid use.^27^ We did not assess the insulin secretion index. In contrast to insulin sensitivity, changes in insulin secretion are associated with the administration of a calcineurin inhibitor, which was not adjusted differently between the two groups.^28^ Therefore, our findings conclusively reveal that only 6 months of SW affected glucose metabolism.

Our findings suggest that a significant improvement in cholesterol levels can be achieved by withdrawing steroids 6 months after transplantation. Dyslipidemia occurs frequently in KT recipients, and about 41% of KT recipients are on statin treatment.^29^ A previous meta‐analysis study indicated that dyslipidemia treatment with statins provides a cardiovascular benefit in KT recipients.^30^ Therefore, lowering total cholesterol and LDL by withdrawing the steroid was beneficial to KT recipients. In our study, total cholesterol, LDL, and HDL significantly decreased in the SW group 12 months posttransplant. In particular, our results show that short‐term SW reduced cholesterol levels. Therefore, if a recipient has stable graft function until at least 6 months posttransplant, we proceed with SW to improve the cholesterol level along with maintaining good graft function. Although cholesterol levels declined in the SW group, other variables, such as BMI and systolic and diastolic blood pressures, were not significantly different between the groups during the study. A longer follow‐up study may be able to reveal the association between SW and these variables. Moreover, a long‐term study is necessary to identify the ultimate effects of a lower cholesterol level, such as the rate of cardiovascular events.

This study had some limitations. First, there was a relatively short follow‐up period. Although 6 months of SW was sufficient to significantly improve glucose and metabolic variables, a longer study is needed to analyze the long‐term effects of SW, such as cardiovascular events. In addition, long‐term analyses of renal graft function in the SW group are needed to more precisely evaluate the safety of SW. A large portion of recipients in the SW group decided to withdrawal based on the clinicians' decision. Although the decision of the clinicians participating in this study did not vary, it might be insufficient to standardize the SW protocol in this study. Furthermore, we could not carry this study without the patient's safety. In other words, we could not withdraw steroids in high‐risk recipients. Finally, the patient's selection criteria at 6 months posttransplant were not an absolute exclusion standard in this study. Many recipients were included in the SC group according to the clinician's judgment, which might include different propensities toward deciding to withdraw or not. We considered that this lack of objectivity is the weakest point of our study. Because our study was a prospective cohort study, not a randomized controlled study, we could not adjust an objective and obvious criteria for reducing immunosuppressive agents in study participants. Further research that has absolute criteria for SW would be necessary to make up for our limitation.

In conclusion, 6 months of SW in recipients with low immunological risk and stable graft function were safely conducted, resulting in improved metabolic profiles. Stable recipients without BPAR and proteinuria can safely withdraw from steroids out of a maintenance immunosuppressive regimen 6 months posttransplant. A long‐term follow‐up study is needed to verify our results.

## CONFLICT OF INTERESTS

The authors declare that there are no conflict of interests.

## AUTHOR CONTRIBUTIONS

Jun B. Bang analyzed the data and wrote the draft. Jun B. Bang, Sang Ho Lee, and Ja Y. Jeon revised the draft and approved the final version of the manuscript. Jun B. Bang, Chang‐Kwon Oh, Yu S. Kim, Sung H. Kim, Hee C. Yu, Chan‐Duck Kim, Man Ki Ju, Byung J. So, Sang Ho Lee, Sang Y. Han, Cheol W. Jung, Joong K. Kim, and Hyung J. Ahn, and participated in designing the study and collecting data.

## Data Availability

The data that support the findings of this study are available from the corresponding author upon reasonable request.
